# Supplementary material for: Rapid laccolith intrusion driven by explosive volcanic eruption
Source: Nat Commun. 2016 Nov 23;7:13585. doi: 10.1038/ncomms13585 (PMC5123016; doi:10.1038/ncomms13585)
Supplement: Supplementary Information — Supplementary Figures 1-8 and Supplementary References. [file ncomms13585-s1.pdf]

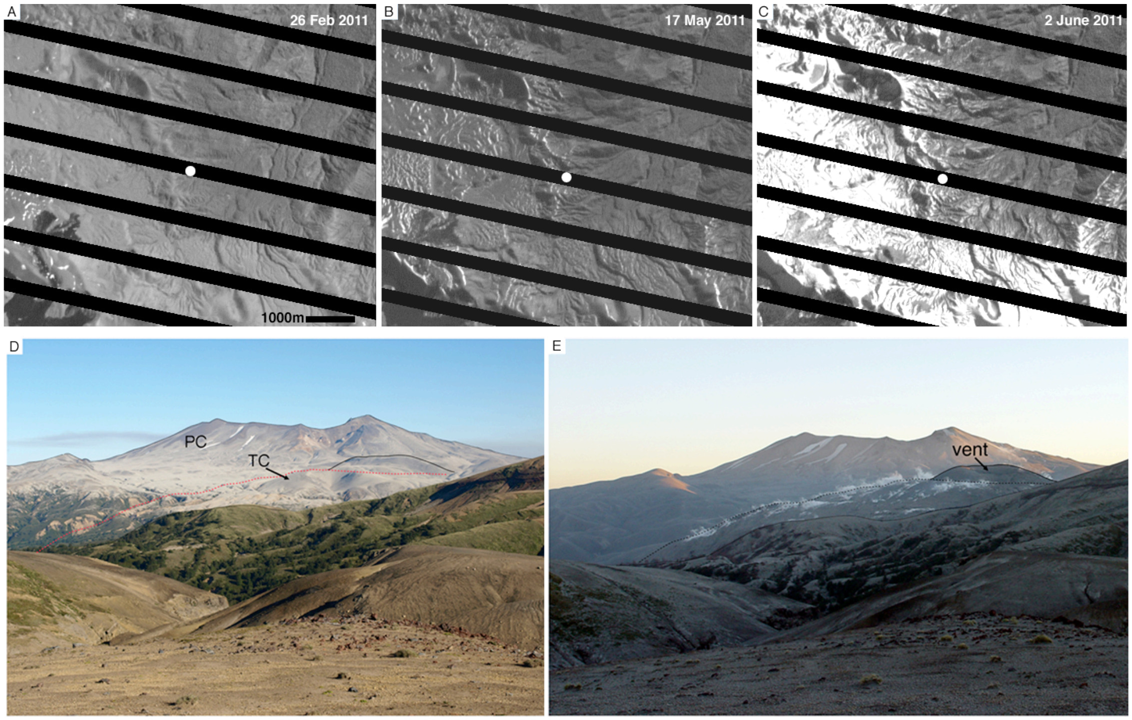

Supplementary Figure 1. Satellite and field imagery of the 2011 Cordón Caulle vent. (A-C) Detailed analysis of a chronology of satellite images obtained from the Landsat 7 mission indicates that at as of 2 June 2011, there was no prominent uplift in the area that would become the vent (white filled circle). The diagonal black swaths are gaps in data due to the 2003 failure of the satellite's scan line corrector. Frames D and E are a comparison of photographs of the (D) pre- and (E) post-eruptive landscape around the active vent (photo in D taken on 18 February 2013 by Mats Landgren; E taken in January 2012 by Jonathan Castro). "PC" indicates Puyehue volcano to the southwest in the background. Photo comparison shows that a large part of the near vent surface has been significantly uplifted such that there is now a broad dome where there was once a highly incised drainage basin. A clear straight-line view to the tuff cone (TC) was possible from the photo vantage point, approximately 3 km to the north of the new (2011) vent, however after the eruption, due to extremely acute uplift in the opening weeks of June 2011, the view became obscured (E). This zone of deformation, provided as a red-dashed overlay in D and highlighted in frame E by fractured ground, hot temperatures, and persistent degassing and fumarolic activity, is the result of more than 175 m of uplift.

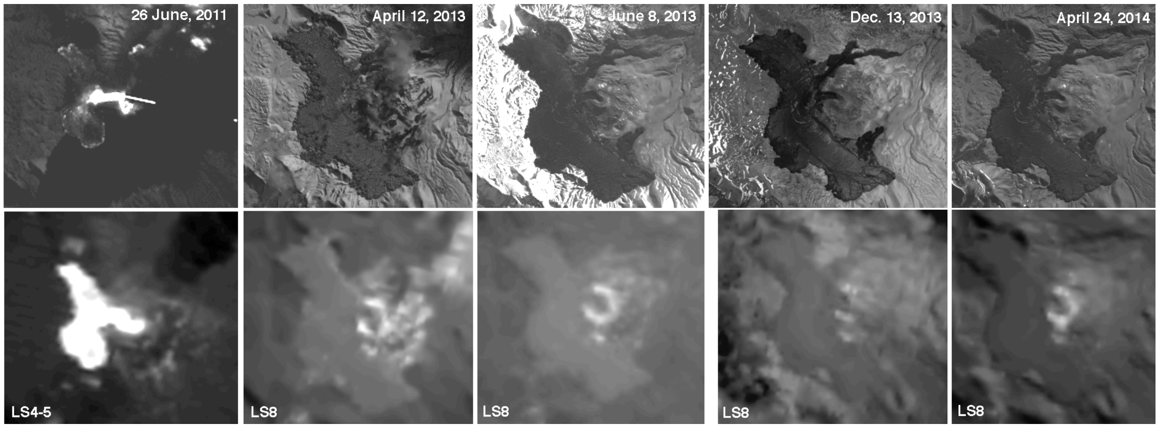

Supplementary Figure 2. Landsat imagery of Cordón Caulle. A series of Landsat 8 optical (upper row) and TIR (bands 11, 12; lower row) images showing the correspondence of surface deformation and high thermal flux zones (bright white). Field of view in all images is about 7 km x 7 km. Beginning around 26 June 2011, thermal anomalies and deformation features became increasingly prominent (also detected in Radar images; Fig. 2). The obsidian lava flow is shown in the lower left TIR image as a very bright form emanating from the vent. Later thermal imagery shows that even though the flow crusted over and therefore emitted less TIR energy, the deformed zone continued to emit very strong TIR. The horseshoe-shaped ring around the vent in each post 26 June 2011 image marks the trace of the early (4-5 June 2011) vent, which is apparently 300-400 meters in diameter, consistent with early observations that the Plinian eruption started from a vent approximately 400 m in diameter (ref 1).

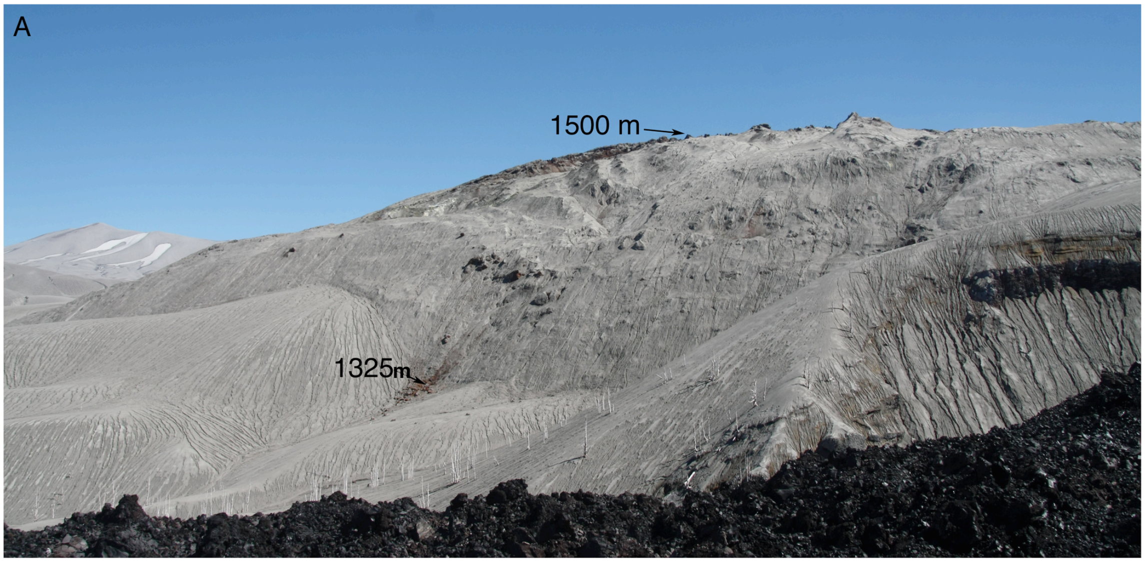

Supplementary Figure 3. Syn-eruptive uplift scarp at Cordón Caulle. (A) Post-eruptive photo of the deformation zone highlighting extreme uplift on the northeastern edge of Cordón Caulle (taken on 11 January 2013 by Hugh Tuffen). The view is to the south. Dark rubble in the foreground is the 2011 lava flow. Dark sub-horizontal outcrop and overlying orange units at the right hand side are pre-eruption lava and tephra deposits respectively; these constitute the geological overburden. Sub-vertical face of the land surface at center field of view is a growth fault, with offset of at least 175 m as evidenced by the difference in DTM derived elevations, shown as numbers on photograph.

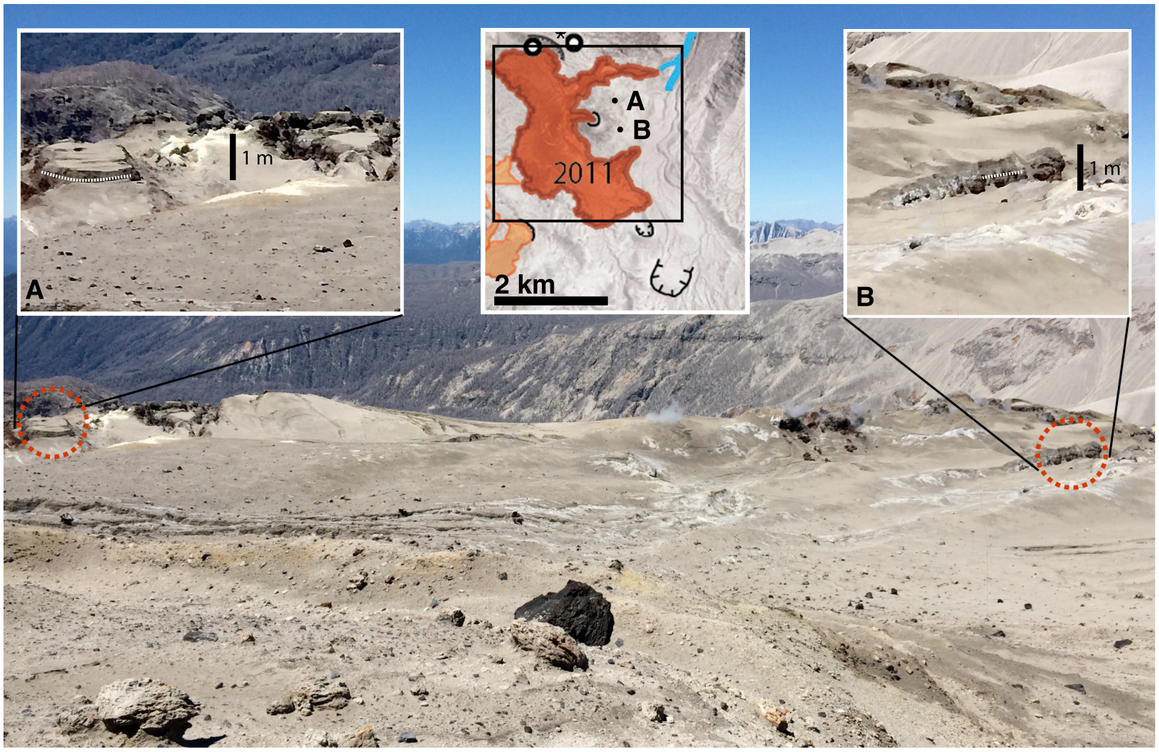

Supplementary Figure 4. Tephra deposit on uplift zone. Field photographs taken from the vent looking east showing the relatively thin (~1m) tephra fall deposit in the proximal area. Small map photos show enlargements of the points in which the 2011 tephra fall deposit is clearly demarcated ("A" and "B" on the inset location map) from subjacent rocks. These tephra deposits are still thinner than the maximum thickness (~4 m) measured in isopach mapping by refs. 2 and 3.

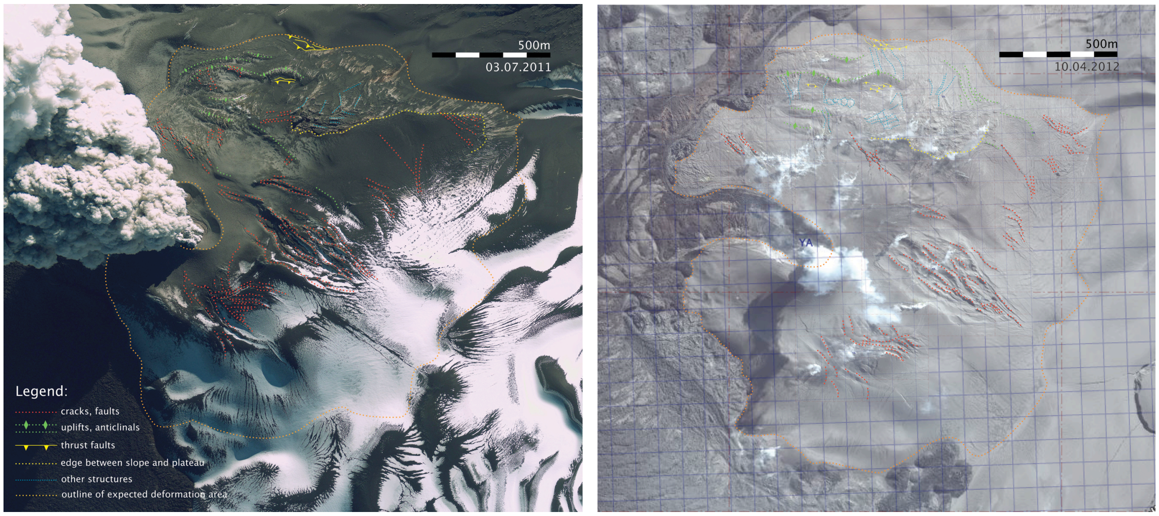

Supplementary Figure 5. Complex surface deformation over the laccolith. (A and B) Post-eruption optical satellite photos showing syn-eruptive deformation field at different times (courtesy of Geoeye Imagery). Images are equivalently scaled and each blue square in (B) represents 100 m horizontal distance. Images show the distribution of several deformation features mapped on images from 3 July 2011 and 10 April 2012 on the zone of uplift. Extensional cracks (red) appear concentrated in the southern and eastern parts of the uplift area, whereas shortening structures including thrust fault and anticlines are apparent in the northern sector. The 175 m “scarp” in the NE quadrant (bold white dashed line) represents a major structure that is best characterized as a growth fault (shown in side view in Supplementary Fig. 3), however is not related to purely extensional or compressive deformation but rather vertical offset. This fault was absent on 6 June 2011 (see radar images in Fig. 2 of main manuscript) and thus must have formed and continued to grow after that date. Even though the vent architecture changed in the intervening time (ie., b/t 03.07.2011 and 10.04.2012), the overall aerial distribution of deformation is approximately constant post 3 July 2011.

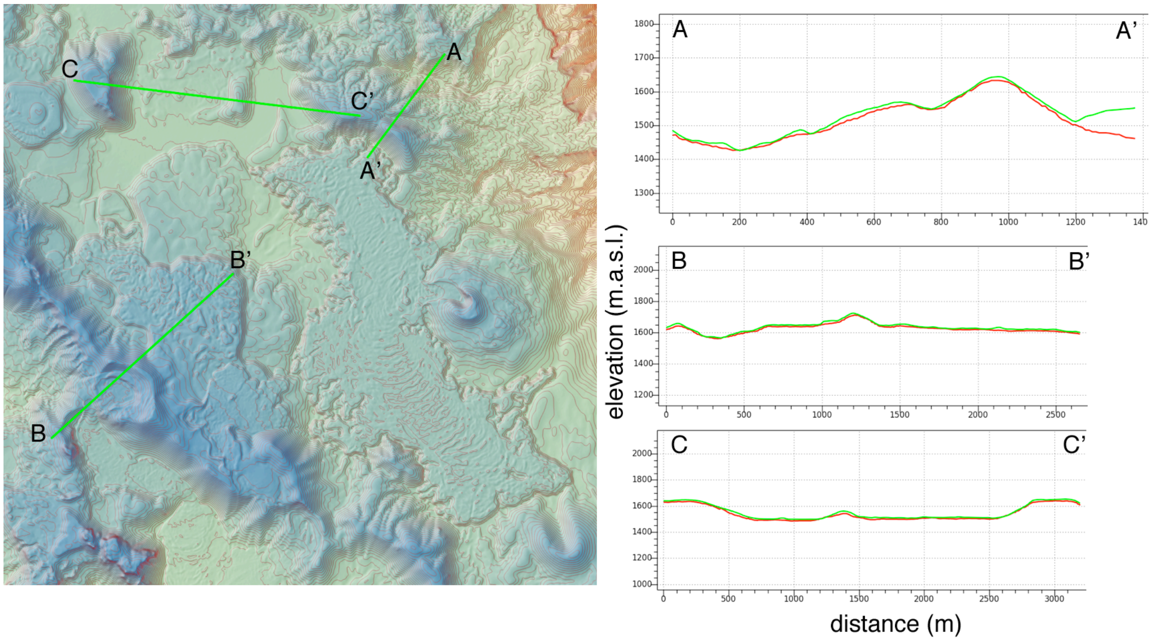

Supplementary Figure 6. Pre- and post-uplift far-field topographic profiles. Topographical profile comparisons in the far field to demonstrate the closeness of elevation data derived from the ASTER global and Pléiades DTMs. The red lines show the pre-eruption ASTER data while green lines are the Pléiades DTM. Closeness of profiles indicates that: a) deformation is absent in the far field, and b) with the exception of the RHS of profile A-A', where the lava flow crosses the profile line, the profiles compare exceptionally well. The vertical and horizontal position error is on the order of 4 m.

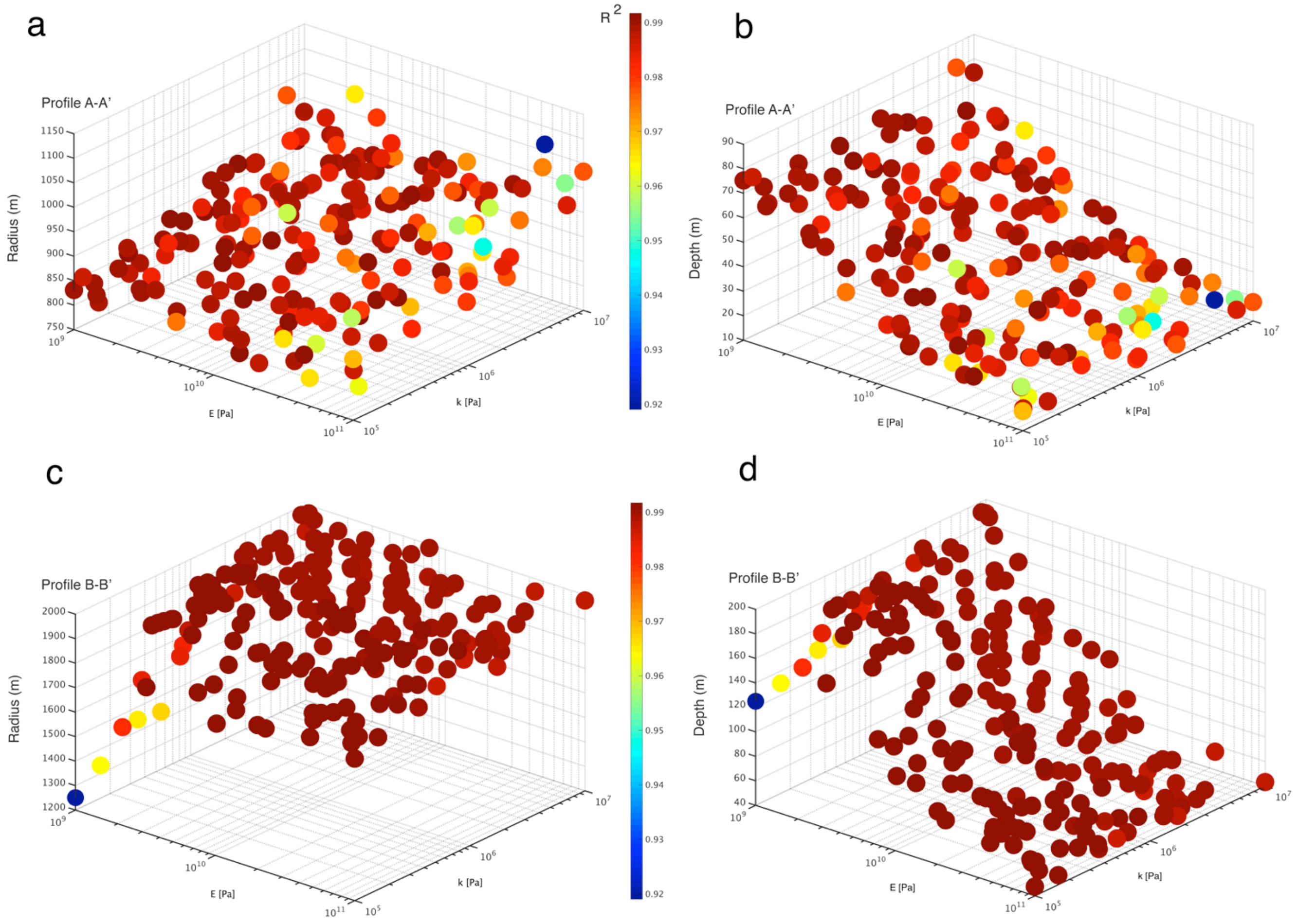

Supplementary Figure 7. Model solution dependency on Young's modulus and elastic foundation thickness. Plot of 200 model solutions for the bending plate on elastic foundation intrusion model of ref 4. Plots on the left show solutions for intrusion radius while those on the right indicate depth of intrusion based on two modeled profiles (A-A' and B-B'; Fig. 4 in manuscript). All models shown here are for and intrusion pressure of 1 MPa. Higher intrusion pressures (up to 10 MPa) shift all depths to greater values. The goodness of fit to natural topography along profiles A-A' and B-B' (e.g., Fig. 4) are shown here as a series of colors, scaling with R^2 values. Most models yield exceptional fits to natural topography indicating firstly, that the interpretation of a shallow laccolithic body being emplaced beneath Cordón Caulle is a valid one, and secondly a range of possible intrusion depths and sizes are possible (~20-200 m and 800-2000 m respectively), depending on the selection of Young's modulus of the overburden (E) and elastic foundation thickness (k).

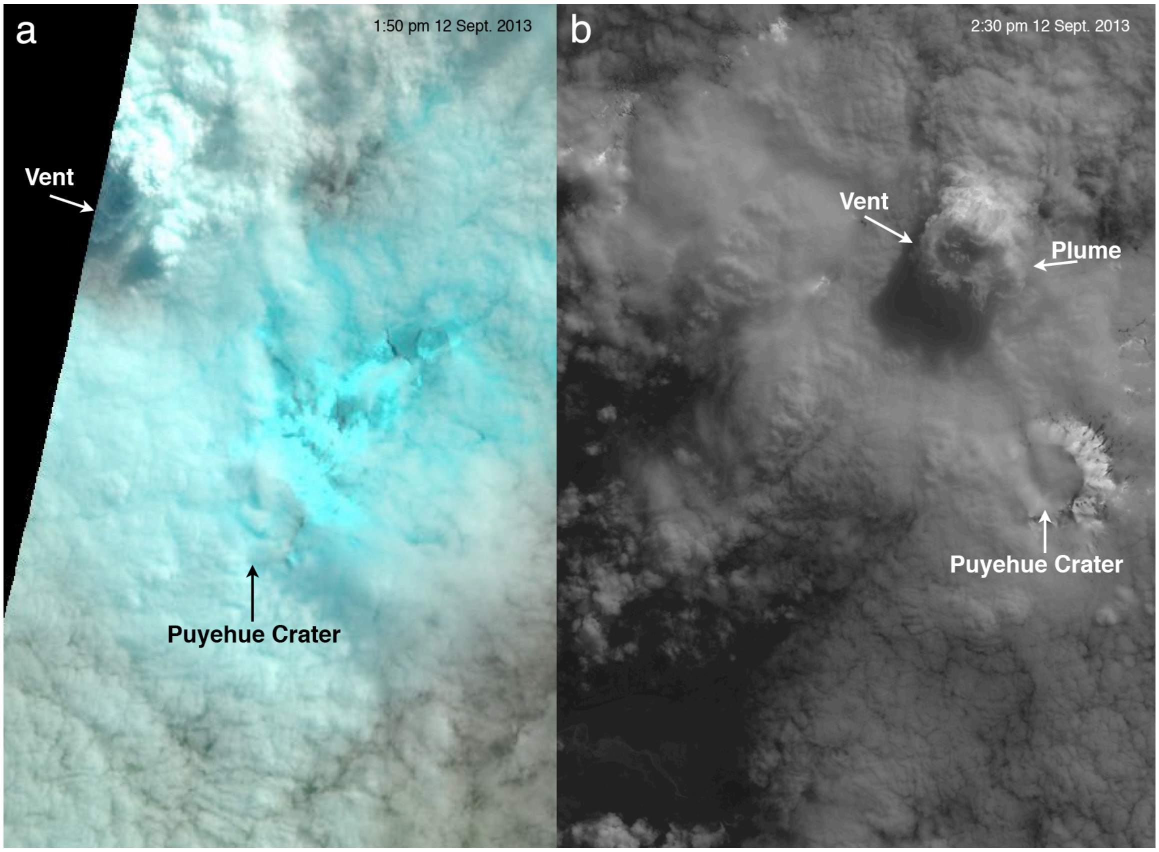

Supplementary Figure 8 Post-eruptive satellite images of the Puyehue-Cordón Caulle complex showing a small explosive eruption on 12 September 2013. The ~2.5 km diameter Puyehue Crater is partially visible through the cloud cover. A) Depicts an image from NASA's EOS-Ali instrument, which shows the onset of the eruption, evidenced by a tall white plume above the vent. B) Is a Landsat 8 visible light image showing the same scene about 1 hour later (2:30 pm local time) when the plume grew in lateral extent and appears to form a more circular column characteristic of a sustained pryoclastic or phreato-magmatic eruption.

References

1. SERNAGEOMIN/OVDAS Puyehue-Cordón Caulle: Reporte especial de actividad volcánica No 38-49 (2011).

2. Pistolesi, M., Cioni, R., Bonadonna, C., Elissondo, M., Boumann, V., Bertagnini, A., Chiari, L., Gonzales, R., Rosi, M., & Francalanci, L. Complex dynamics of small-moderate volcanic events: the example of the 2011 rhyolitic Cordón Caulle, eruption, Chile. *Bull. Volcanol.* **77**, 3-27 (2015).

3. Silva Parejas, C., Lara, L.E., Bertin, D., Amigo, A., & Orozco, G. The 2011-2012 eruption of Cordón Caulle volcano (Southern Andes): evolution, crisis management and current hazards. *EGU Gen. Assem.* **9382** (2012).

4. Galland, O., & Scheibert, J. Analytical model of surface uplift above axisymmetric flat-lying magma intrusions: Implications for sill emplacement and geodesy. *J. Volcanol. Geotherm. Res.* **253**, 114-130 (2013).
